# Human Fecal Microbiota Transplantation Reduces the Susceptibility to Dextran Sulfate Sodium-Induced Germ-Free Mouse Colitis

**DOI:** 10.3389/fimmu.2022.836542

**Published:** 2022-02-14

**Authors:** Yapeng Yang, Xiaojiao Zheng, Yuqing Wang, Xiang Tan, Huicong Zou, Shuaifei Feng, Hang Zhang, Zeyue Zhang, Jinhui He, Bota Cui, Xueying Zhang, Zhifeng Wu, Miaomiao Dong, Wei Cheng, Shiyu Tao, Hong Wei

**Affiliations:** ^1^ College of Animal Science and Technology, Huazhong Agricultural University, Wuhan, China; ^2^ Center for Translational Medicine, Shanghai Key Laboratory of Diabetes Mellitus and Shanghai Key Laboratory of Sleep Disordered Breathing, Shanghai Jiao Tong University Affiliated Sixth People’s Hospital, Shanghai, China; ^3^ Medical Center for Digestive Diseases, The Second Affiliated Hospital of Nanjing Medical University, Nanjing, China; ^4^ Intestinal Microenvironment Treatment Center, Tenth People’s Hospital of Tongji University, Shanghai, China

**Keywords:** fecal microbiota transplantation, inflammatory bowel disease, germ-free mice, metagenomic, metabolomics

## Abstract

In clinical practice, fecal microbiota transplantation (FMT) has been used to treat inflammatory bowel disease (IBD), and has shown certain effects. However, the selection of FMT donors and the mechanism underlying the effect of FMT intervention in IBD require further exploration. In this study, dextran sodium sulfate (DSS)-induced colitis mice were used to determine the differences in the protection of colitis symptoms, inflammation, and intestinal barrier, by FMT from two donors. Intriguingly, pre-administration of healthy bacterial fluid significantly relieved the symptoms of colitis compared to the ulcerative colitis (UC) bacteria. In addition, healthy donor (HD) bacteria significantly reduced the levels of inflammatory markers Myeloperoxidase (MPO) and Eosinophil peroxidase (EPO), and various pro-inflammatory factors, in colitis mice, and increased the secretion of the anti-inflammatory factor IL-10. Metagenomic sequencing indicated higher species diversity and higher abundance of anti-inflammatory bacteria in the HD intervention group, including *Alistipes putredinis*, *Akkermansia muciniphila*, *Bifidobacterium adolescentis*, short-chain fatty acids (SCFAs)-producing bacterium *Christensenella minuta*, and secondary bile acids (SBAs)-producing bacterium *Clostridium leptum*. In the UC intervention group, the SCFA-producing bacterium *Bacteroides stercoris*, IBD-related bacterium *Ruminococcus gnavus*, *Enterococcus faecalis*, and the conditional pathogen *Bacteroides caccae*, were more abundant. Metabolomics analysis showed that the two types of FMT significantly modulated the metabolism of DSS-induced mice. Moreover, compared with the UC intervention group, indoleacetic acid and unsaturated fatty acids (DHA, DPA, and EPA) with anti-inflammatory effects were significantly enriched in the HD intervention group. In summary, these results indicate that FMT can alleviate the symptoms of colitis, and the effect of HD intervention is better than that of UC intervention. This study offers new insights into the mechanisms of FMT clinical intervention in IBD.

**Graphical Abstract:**
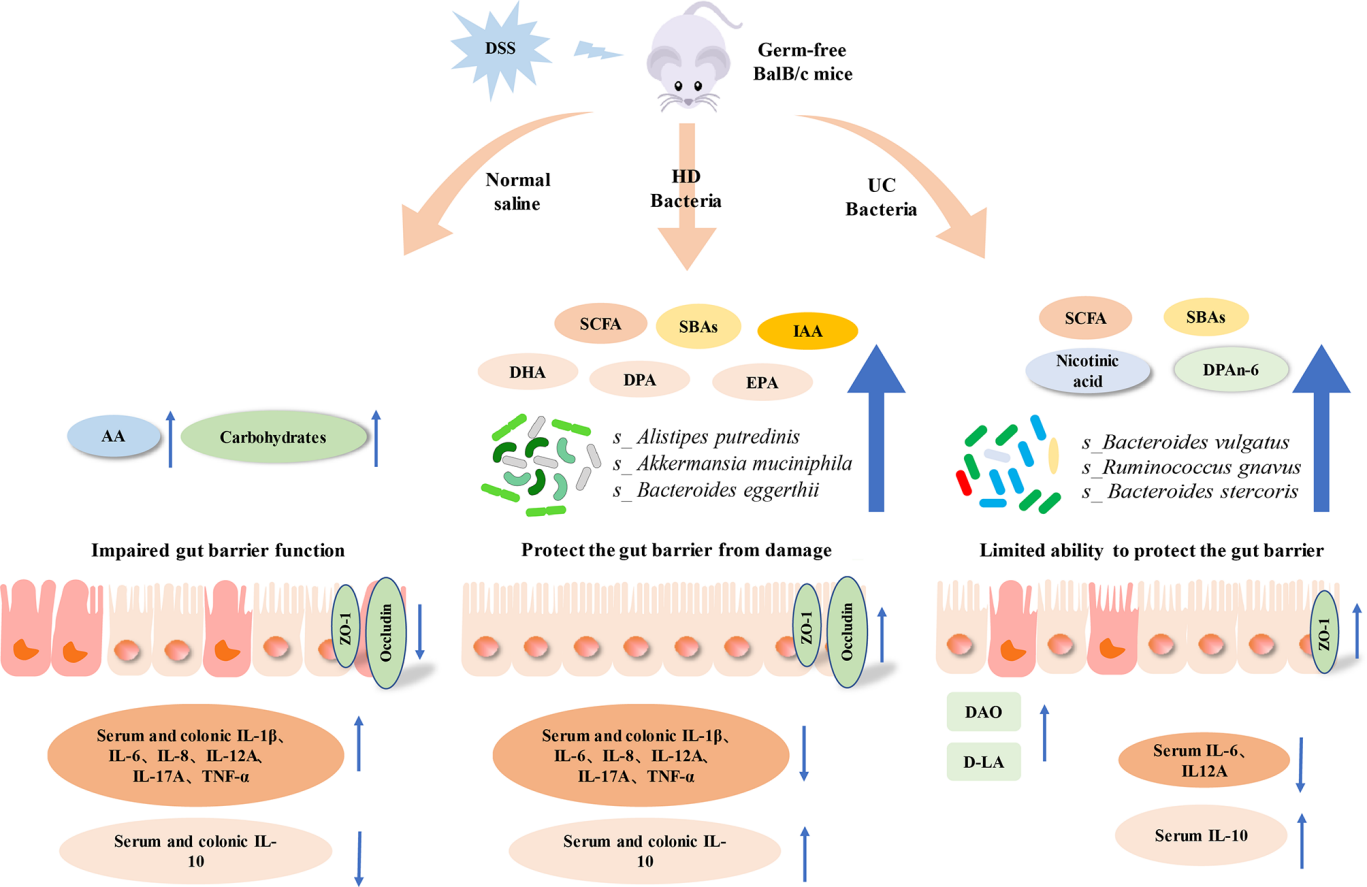
DSS, Dextran Sulfate Sodium; HD, Healthy donor; UC, Ulcerative colitis; AA, Amino acid; SCFA, Short chain fatty acid; SBA, Secondary bile acid; IAA, Indoleacetic acid; DHA, Docosahexaenoic acid; DPA, Docosapentaenoic acid; EPA, Eicosapentaenoic acid.

## Introduction

Inflammatory bowel disease (IBD) is a complicated and chronic intestinal disease, consisting of Crohn’s disease (CD) and ulcerative colitis (UC) ( 1 ). IBD has been reported to be influenced by genetics, environment, and intestinal microbes ( 2–4 ), and is characterized by host immune response, destruction of the intestinal barrier, and changes in the intestinal microbial community, accompanied by intestinal bleeding, diarrhea, and weight loss ( 5, 6 ). In IBD patients, antigen invasion can activate the mucosal immune response, causing macrophages to release pro-inflammatory cytokines such as IL-1β, IL-6, IL-8, and TNF-α, and thus aggravate inflammation ( 7 ). UC mainly affects the superficial layer of colonic mucosa, and histological analysis shows mucosal ulceration and a large amount of inflammatory cell infiltration, which is characterized by an increase in the numbers of CD4^+^ T lymphocytes, neutrophils, and eosinophils ( 8, 9 ). Unfortunately, IBD seriously affects the quality of life and increases the risk of colon cancer ( 10 ).

The intestinal microbiota is involved in immune system development and other processes, and imbalance in intestinal microecology is closely associated with the occurrence and development of a variety of human diseases ( 11 ), including IBD ( 12 ). The gut microbiota composition and metabolism of IBD patients are significantly different when compared with those of healthy people ( 13, 14 ), and is characterized by a decrease of *Faecalibacterium prausnitzii* and *Roseburia hominis* in patients with UC ( 15 ). In addition, the levels of short-chain fatty acids (SCFAs), secondary bile acids (SBAs), indole acetic acid (IAA), and other metabolites that have anti-inflammatory effects, are also significantly reduced in IBD ( 16 ). Therefore, methods that target intestinal microbes and their metabolites have potential applications in IBD treatment ( 16 ).

Fecal microbiota transplantation (FMT) refers to the transplantation of functional flora from healthy human feces into the intestinal tract of patients, rebuilding new intestinal flora, and thus treating diseases ( 17 ). Clinical trials have shown that FMT is effective for treating UC ( 18, 19 ). There were also animal experiments to investigate the therapeutic mechanism of FMT in the treatment of IBD. Marina Lleal et al. found that FMT reduced disease severity by increasing the relative abundance of probiotic microbes ( 20 ). Healthy human fecal samples rich in Firmicutes downregulated the TH17 pathway and reduced inflammation of colon tissues in mice ( 21 ). However, as yet, we do not understand the mechanism of action of FMT, and its causal relationship with the treatment of diseases ( 21 ). Further research is necessary to identify the microbes that are responsible for the therapeutic effect of FMT, in order to develop more effective microbiota based treatments for UC ( 18, 22 ).

As a model with a clear floral background, germ-free (GF) mice can be used to study the causal relationship between human intestinal flora and host health or disease by constructing humanized flora mice (HFA mice) ( 23 ). At present, there are a few studies using HFA mice to study the effect of human donor bacteria on the sensitivity of DSS-induced acute colitis. In this study, we hypothesized that GF mice hosting human flora can maintain cytokine homeostasis and improve the intestinal barrier through anti-inflammatory bacteria and their metabolites, thereby reducing the sensitivity of DSS-induced colitis. Given that the intestinal microbiota and its metabolites play a key role in relieving IBD, we first introduced the healthy donor (HD) and UC microbiota in GF mice, induced colitis using DSS, and analyzed mouse serum and colon immune homeostasis and intestinal epithelial barrier function, to understand the impact of the two different donor treatments on colitis. In addition, the differences in the intestinal microbial composition and metabolism between the two donor-treated groups of mice were compared, to provide targets and directions for clinical IBD treatment from the perspective of intestinal microecology.

## Materials and Methods

### FMT Preparation

Healthy donor fecal bacteria suspension comes from the Chinese fmtBank (Nanjing, China), UC donor stool comes from Shanghai Tenth People’s Hospital, fecal samples from 3 patients with active UC were mechanically homogenized in equal amounts under anaerobic condition, and the mixed feces were added into sterile normal saline and glycerol mixed solution (ratio of saline and glycerol = 85:15, Per 100 ml) at a ratio of 10%(w/v), roughly filtered through sterile gauze, and then filtered through a sterile 100μm filter to prepare fecal bacteria suspension ( 24 ).

### Animals and Treatments

**Figure 1:**
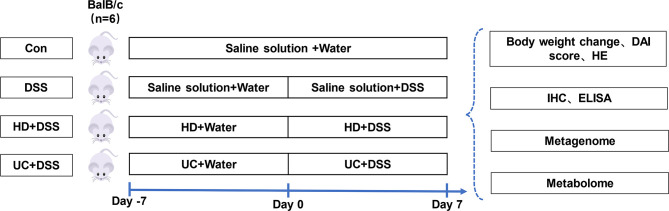
Study design.

8-10 weeks GF BalB/c female mice were obtained from the GF animal platform of Huazhong Agricultural University. Mice were housed in a pathogen-free colony (temperature, 25 ± 2°C; relative humidity, 45%-60%; lighting cycle, 12 h/day; light hours 06:30-18:30) with free access to food and water. To investigate the effect of FMT with different donors on IBD, mice were split into the Control, DSS, Healthy Donor intervention group (HD+DSS) and Ulcerative Colitis intervention group (UC+DSS). Two FMT groups were first colonized with bacterial suspension for 7 days (100μL/d), and then mice were treated with 3% DSS solution for 7 days, while the bacterial solution continued to intervene, and then samples were taken. The experimental grouping and timeline are shown in **Figure 1**. The body weight was recorded daily during the experiment, and the difference between the body weight on the day of measurement and that on day 0 was calculated (25). All experimental methods in this study were carried out following the Guide for the Care and Use of Laboratory Animals at Huazhong Agricultural University. The animal experiment ethics number for this study is HZAUMO-2021-0183.

### Disease Activity Index

The disease activity index (DAI) score includes weight loss, stool consistency, and stool bleeding. Refer to the **Table 1** below for detailed scores (26, 27). In brief, DAI was determined by an investigator blinded to the protocol by scoring changes in weight (no change = 0; 1-5% = 1; 5-10% = 2; 10-15% = 3; >15% = 4), Bloody stool score (Normal colored stool = 0; Brown stool = 1; Reddish stool = 2; Bloody stool = 3; Gross bleeding = 4), and stool consistency (Normal stools, well-formed pellets = 0; loose stools,pasty stool that does not stick to the anus =1-2; and diarrhea, liquid stools that sticks to the anus =3-4).

**Table 1:** Disease activity index.

Score	Weight loss (%)	Stool consistency	Bloody stool score
0	None	Normal	Normal colored stool
1	1-5	Loose stool	Brown stool
2	5-10	Loose stool	Reddish stool
3	10-15	Diarrhea	Bloody stool
4	>15	Diarrhea	Gross bleeding

Disease activity index (DAI), mean score of weight loss, stool consistency, and bloody stool score.

### Histologic Analysis of Mice Colon

The distal colon of the mouse was fixed with 4% paraformaldehyde, embedded in paraffin, and cut into 4 μm thick sections. The sections were stained with hematoxylin-eosin (HE), Periodic Acid-Schiff (PAS), Alcian Blue, and immunohistochemistry (IHC). Intestinal tissue damage was scored as previously described (**Table 2**) (28). According to the manufacturer’s instructions, the expressions of colonic tight junction proteins ZO-1(Proteintech Group, Inc. 21773-1-AP) and Occludin (Protein Tech Group, Inc. 27260-1-AP) were detected by IHC, and the average optical density (AOD) was statistically analyzed by Image Pro Plus 6.0 (Media Cytology, Inc.). Images were collected under a microscope (Nikon eclipse 80i, Japan) or high resolution slide scanning system (Pannoramic MIDI, Hungary 3DHISTECH Ltd).

**Table 2:** Histological grading of colitis.

Grade	Inflammation	Extent	Crypt damage	Percent involvement
0	None		None	0
1	Slight	Mucosa	Basal 1/3 damage	1%-33%
2	Moderate	Mucosa and Submucosa	Basal 2/3 damage	34%-66%
3	Severe	Transmural	Entire crypt and epithelium lost	67%-100%

### Enzyme-Linked Immunosorbent Assay

All measurements are performed in accordance with the manufacturer’s instructions. Concentrations of IL-1β (ml063132), IL-6 (ml002293), IL-8 (ml001856), IL-10 (ml002285), IL-12A (ml157145), TNF-α (ml002095), IL-17A (ml037864), Foxp3 (ml037859), myeloperoxidase (MPO) (ml002070), eosinophilic peroxidase (EPO) (ml769125) and diamine oxidase (DAO) (ml002199) were determined in the serum and (or) colon samples using ELISA kits (Shanghai Enzyme-linked Biotechnology, Shanghai, China), The absorbance (OD value) was measured with a microplate (BioTek Instruments, Inc) reader at 450nm, and the cytokine content in the sample was calculated from the standard curve. Serum D-lactic acid (D-LA) (ml158174) content was measured by D-Lactic acid detection kit (Shanghai Enzyme-linked Biotechnology, Shanghai, China).

### Fecal Microbiota Analysis

Once the mouse feces samples were gathered in a clean cage inside the isolator, the samples were shipped with dry ice for DNA extraction (Cetyltrimethylammonium Ammonium Bromide, CTAB) and sequencing. A total amount of 1μg DNA per sample (OD value is between 1.8~2.0) was used as input material for the DNA sample preparations. Sequencing libraries were generated using NEBNext^®^ Ultra™ DNA Library Prep Kit for Illumina (NEB, USA) following manufacturer’s recommendations and index codes were added to attribute sequences to each sample. Briefly, the DNA sample was fragmented by sonication to a size of 350bp, then DNA fragments were end-polished, A-tailed, and ligated with the full-length adaptor for Illumina sequencing with further PCR amplification. At last, PCR products were purified (AMPure XP system) and libraries were analysed for size distribution by Agilent2100 Bioanalyzer and quantified using real-time PCR. The clustering of the index-coded samples was performed on a cBot Cluster Generation System according to the manufacturer’s instructions. After cluster generation, the library preparations were sequenced on an Illumina HiSeq platform and paired-end reads were generated. Utilizing MetaPhlAn3 (version3.0.7) software, the reads of the filtering host was compared with the Chocophlan (Version MPa _ V30 _ Chocophlan _ 201901) database to obtain the species abundance information for further analysis. The data were imported into Rstudio(4.1.1) and plotted using ggplot2(3.3.5) for the mapping of species composition, α diversity, principal coordinate analysis (PcoA), and LEfSe(LDA>2, light red for HD_DSS group, and light blue for UC_DSS group). Differential bacteria and phenotypic data were combined according to the groups and imported into Rstudio(4.1.1) to calculate the correlation coefficient R and P value using cor and RCC functions. The data were imported into Rstudio to draw the phenotypic correlation heat map using Phenmap package (1.0.12), where **p< 0.01, *p < 0.05.

### Metabolome Analysis

Metabolomics analysis on fecal samples was conducted using the Q300 Metabolite Assay Kit (Human Metabolomics Institute, Inc., Shenzhen, Guangdong, China) based on the method previously published with modifications ( 29 ). Briefly, 10mg of each freeze-dried sample was accurately weighed, homogenized by 50ul of water, and extracted by 240ul of methanol. The sample mixture was then centrifugation for 20min. An aliquot of 5ul supernatant was added to a 96-well plate and derivatized according to the manufacturer’s instruction. After sample preparation, the plate was sealed for analysis. An ultra-performance liquid chromatography (UPLC, Agilent 1290 Infinity, USA) coupled to tandem mass spectrometry (MS, Agilent 6460 Triple Quad, USA) was used to quantitatively measure the metabolites. The raw data files generated by UPLC-MS/MS were processed using the TMBQ software (v1.0, Human Metabolomics Institute, Inc., Shenzhen, Guangdong, China) to perform peak integration, calibration, and quantitation for each metabolite. The differential metabolites can be obtained using univariate statistical analysis (Anova or Kruskal-Wallis test, depending on the normality of data and homogeneity of variance). In this project, threshold value for differential metabolites selection is: P < 0.05. For the potential biomarkers of the DSS group, HD+DSS group and UC+DSS group, screening criteria are as follows: P < 0.05 and |log2FC| >= 0 in univariate statistics analysis and Variable importance in projection (VIP) > 1 in multi-dimensional statistics.

### The Co-Occurrence Analysis

The co-occurrence among Bacteria, Immunity, Biomarker metabolite, Barrier was calculated on the basis of the relative abundance by Spearman’s rank correlation coefficient (P < 0.05) used R package Hmisc (version 4.5-0). The network layout was calculated and visualized using a circular layout by the Cytoscape software. Only edges with correlations greater than 0.5 were shown in the two nodes, and unconnected nodes were omitted. Correlation coefficients with a magnitude of 0.5 or above were selected for visualization in Cytoscape (version 3.8.2).

### Statistical Methods

The data were analyzed using GraphPad Prism 6 (GraphPad Software, San Diego, CA). Student T Test or Mann-Whitney U Test was selected for statistical analysis between two groups depending on the normality of data and homogeneity of variance, Data from more than two groups were compared using one-way ANOVA followed by Tukey’s multiple comparison tests, All data were represented as means ± SEM. P ≤ 0.05 was considered statistically significant.

## Results

### HD Microbiota Alleviated DSS-Induced GF Mice Acute Colitis

**Figure 2:**
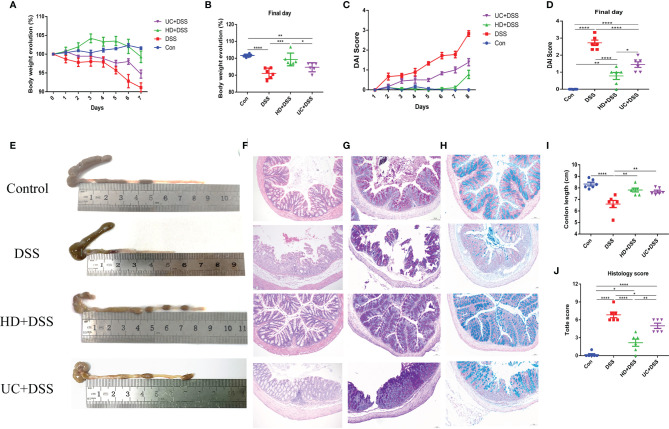
Oral administration of human microbiota alleviated DSS-induced acute colitis symptoms of GF mice. **(A)** Body weight evolution; **(B)** Body weight change in the final day; **(C)** DAI score; **(D)** DAI score in the final day; **(E)** Representative colon; **(F)** Colon length; **(G)** HE staining of colon tissue (100×); **(H)** Histological score; **(I)** PAS staining (100μm, n = 3); **(J)** Alican blue staining (100μm, n = 3). *p ≤ 0.05, **p ≤ 0.01, ***p ≤ 0.001, ****p ≤ 0.0001, data are represented as mean ± SEM.

To investigate the effect of FMT from the two donors on IBD, DSS-induced colitis mice were administered an HD bacterial solution, UC bacterial solution, or normal saline. Treatment with 3% DSS led to considerably reduced body weight in mice, and this loss was alleviated by HD bacterial solution to a greater extent than the UC bacterial solution **(** **Figures 2A, B** **)**. High DAI induced by DSS was significantly decreased by HD and UC interventions, and the DAI in the HD+DSS group was significantly lower than that in the DSS and UC+DSS groups **(** **Figures 2C, D** **)**. In addition, the length of the colon was restored by FMT **(** **Figures 2E, I** **)**. Histological staining results showed that DSS treatment caused varying degrees of histological damage in each group of mice. Compared to the DSS only group, FMT reduced inflammatory cell infiltration, prevented mucosal damage of colon tissue, and decreased the histological score. Compared to the UC+DSS group, the HD+DSS group showed greater reduction of the damage to colon tissue caused by DSS **(** **Figures 2F–H, J** **)**. In summary, these results indicated that bacteria derived from healthy people or UC patients could alleviate DSS-induced colitis, and the intervention efficacy of the HD bacterial solution was significantly better than that of the UC bacterial solution.

### Effects of FMT on Colonic Inflammatory Markers

**Figure 3:**
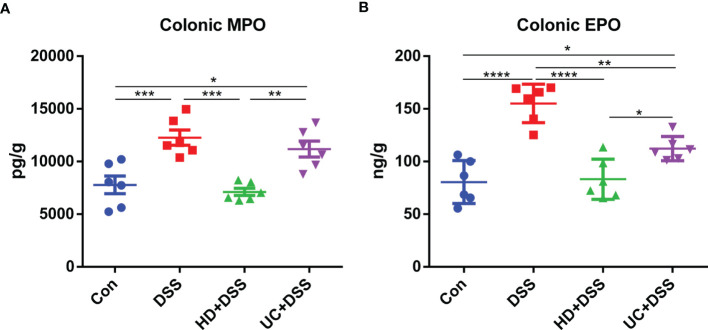
HD intervention significantly reduced the levels of two markers in DSS-induced colitis. **(A)** Colonic MPO; **(B)** Colonic EPO. *p ≤ 0.05, **p ≤ 0.01, ***p ≤ 0.001, ****p ≤ 0.0001, data are represented as mean ± SEM.

We next evaluated the effect of FMT on colonic inflammatory markers MPO and EPO, using ELISA. As shown in **(** **Figures 3A, B** **)**, compared to the DSS and UC+DSS groups, lower levels of MPO and EPO were observed in the colon tissues of HD+DSS group. And while MPO levels were not significantly different between the UC+DSS and DSS groups, UC intervention downregulated the EPO levels. These results indicated that HD intervention could better alleviate the inflammatory response to DSS compared to UC intervention.

### Effects of FMT on Serum and Colonic Cytokines

**Figure 4:**
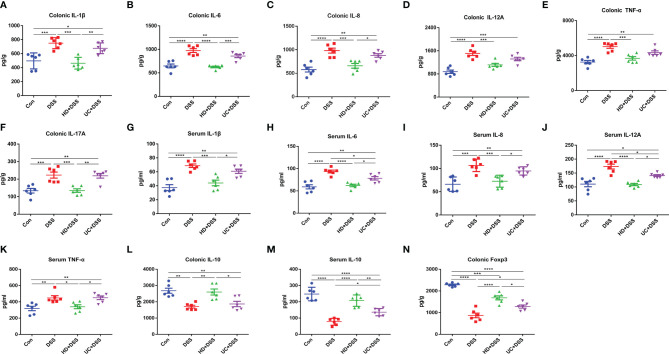
The levels of cytokines in the serum and colon groups. **(A)** Colonic IL-1β; **(B)** Colonic IL-6; **(C)** Colonic IL-8; **(D)** Colonic IL-12A; **(E)** Colonic TNF-α; **(F)** Colonic IL-17A; **(G)** Serum IL-1β; **(H)** Serum IL-6; **(I)** Serum IL-8; **(J)** Serum IL-12A; **(K)** Serum TNF-α; **(L)** Colonic IL-10; **(M)** Serum IL-10; **(N)** Colonic Foxp3. *p ≤ 0.05, **p ≤ 0.01, ***p ≤ 0.001, ****p ≤ 0.0001, data are represented as mean ± SEM.

Since intestinal immune disorders have been observed in IBD patients, we next explored the effects of HD and UC intervention on the immune homeostasis of cytokines in mice. The levels of pro-inflammatory factors IL-1β, IL-6, IL-6, IL-8, IL-12A, TNF-α, and IL-17A, and anti-inflammatory factor IL-10 in colon tissue and (or) serum, were measured by ELISA. As shown in **Figure 4**, compared to the DSS group, HD+DSS group had significantly reduced the levels of pro-inflammatory factors **(** **Figures 4A-K** **)** and increased IL-10 **(** **Figures 4L, M** **)** levels in colon tissue and (or) serum. UC intervention only reduced IL-6 and IL-12A levels in the serum **(** **Figures 4H, J** **)**. There were higher levels of IL-10 in the serum **(** **Figure 4M** **)** and no significant differences in colon tissue between the UC+DSS and DSS groups **(** **Figure 4L** **)**. In addition, compared to the UC+DSS group, the levels of pro-inflammatory factors were lower and those of IL-10 were higher in the HD+DSS group **(** **Figures 4A-M** **)**, both in serum and colon tissues. We also found that the levels of the transcription factor Foxp3, which controls regulatory T cell development, were significantly higher in the HD+DSS and UC+DSS groups compared to the DSS group, and were higher in the HD+DSS group than in the UC+DSS group **(** **Figure 4N** **)**. Taken together, FMT was found to exert anti-inflammatory effects by maintaining the homeostasis of cytokines, and the effect of HD intervention was better than that of UC intervention.

### HD Intervention Improved Intestinal Barrier in DSS-Induced Colitis Mice

**Figure 5:**
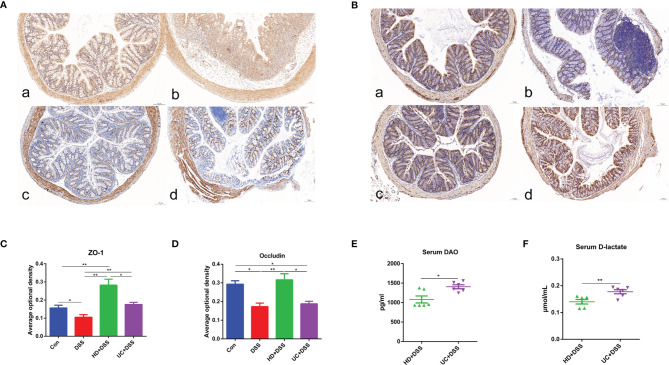
FMT significantly enhances the intestinal barrier of mice. Immunohistochemistry for ZO-1 **(A)** and Occludin **(B)** in each group (100μm, n = 3); **(C, D)** Average optical density. The changes of levels of serum DAO **(E)** and D-lactate **(F)**. a: Control, b: DSS, c: HD+DSS, d: UC+DSS. *p ≤ 0.05, **p ≤ 0.01, data are represented as mean ± SEM.

Since intestinal barrier dysfunction is involved in IBD, we hypothesized that FMT reduces DSS-induced damage by protecting the intestinal barrier. The expression of tight junction proteins ZO-1 and occludin in the four groups was observed by IHC. As shown in **Figures 5A–D**, the AOD of ZO-1 and occludin in the colon of the HD+DSS group was significantly higher than that of the DSS group. Meanwhile, the AOD of colon ZO-1 was significantly higher in the UC+DSS group than in the DSS group. However, the AOD of occludin was not significantly different between the UC+DSS and DSS groups. In addition, the AOD of ZO-1 and occludin in the colonic tissues of HD+DSS group was significantly higher than that in the UC+DSS group. To elucidate the barrier mechanism underlying the difference in the efficacy of FMT intervention in colitis, we measured the concentrations of DAO and D-LA, which are involved in intestinal permeability in serum, after FMT intervention. The results **(** **Figures 5E, F** **)** indicated lower levels of DAO and D-LA in the serum of HD+DSS mice. Taken together, these results indicate that HD intervention reduced DSS-induced damage to a greater extent compared to UC intervention, by ensuring the integrity of the intestinal barrier.

### Microbiota were Significantly Different Between HD+DSS Group and UC+DSS Group

**Figure 6:**
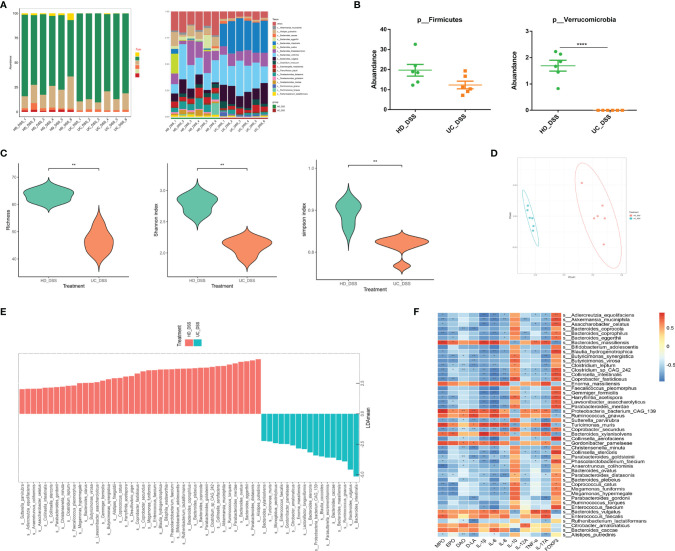
Differences in feces microbes of recipient mice. **(A)** Microbial composition at phylum level and species level; **(B)** Abundance of Verrucomicrobia and Firmicutes; **(C)** Alpha diversity of species composition, Richness, Shannon index, Simpson; **(D)** PcoA; **(E)** LEfSe; **(F)** Heatmap of different microorganisms, cytokines, and intestinal barrier. * p ≤ 0.05, ** p ≤ 0.01, **** p ≤ 0.0001, data are represented as mean ± SEM.

The gut microbiota is essential for the initiation and progression of IBD, and the two donor microbiota showed different efficacies in DSS-induced colitis. Therefore, we analyzed the differences in fecal microbiota between the two groups of recipient mice by metagenomic analysis. The results of microbial composition at the phylum and species levels are shown in **Figure 6A**, and Firmicutes and Bacteroides were found to be dominant in both groups. At the species level, the relative abundances of *Bacteroides eggerthii, Parabacteroides merdae*, and *Akkermansia muciniphila* (AKK), were higher in the HD+DSS group than in the UC+DSS group, while the relative abundances of *Bacteroides intestinalis*, *Bacteroides vulgatus*, and *Bacteroides stercoris* were higher in the UC+DSS group than in the HD+DSS group **(** **Figure 6A** **)**. The relative abundance of Firmicutes was higher in the HD+DSS group than in the UC+DSS group, but the difference was not statistically significant, and Verrucomicrobia only existed in the HD+DSS group **(** **Figure 6B** **)**. As expected, richness, Shannon index, and Simpson’s index, showed that the alpha diversity of intestinal microbes in the HD+DSS group was significantly higher than that in the UC+DSS group **(** **Figure 6C** **)**. In addition, principal coordinate analysis (PCoA) based on Bray-Curtis metric distance showed that the fecal microbiota of the HD+DSS group was significantly different from that of the UC+DSS group **(** **Figure 6D** **)**. Linear discriminant analysis effect size (LEfSe, p < 0.05, LDA > 2.0) was performed to determine the differences in species abundance between the two groups; *B. intestinalis*, *B. vulgatus*, *Ruminococcus gnavus*, and *Enterococcus faecalis*, were more abundant in the fecal microbiota of the UC+DSS group. However, *B. eggerthii*, *Bacteroides ovatus*, *P. merdae*, AKK, *Clostridium leptum*, and *Bifidobacterium adolescentis* were more abundant in the fecal microbiota of the HD+DSS group **(** **Figure 6E** **)**. Heat map showed the correlation between the differential bacteria and cytokines **(** **Figure 6F** **)**. A variety of bacteria enriched in the HD+DSS group were positively correlated with anti-inflammatory factors and/or Foxp3, and negatively correlated with inflammatory markers and/or pro-inflammatory factors and/or indicators of intestinal barrier damage, including Akk*, B. eggerthii*, *C. leptum*, *P. distasonis*, and *P. merdae*. In the UC+DSS group, *B. massiliensis*, *R. gnavus*, *B. xylanisolvens*, and *B. vulgatus*, were enriched and positively correlated with inflammatory markers, pro-inflammatory factors, and intestinal barrier damage indicators, and negatively correlated with anti-inflammatory factors and Foxp3. In summary, microbiota were markedly different between the HD+DSS and UC+DSS groups, and many kinds of bacteria were associated with anti-inflammatory effects in the HD+DSS group.

### FMT Modulates Metabolism in DSS-Induced Colitis and HD Intervention Is More Effective than UC Intervention

**Figure 7:**
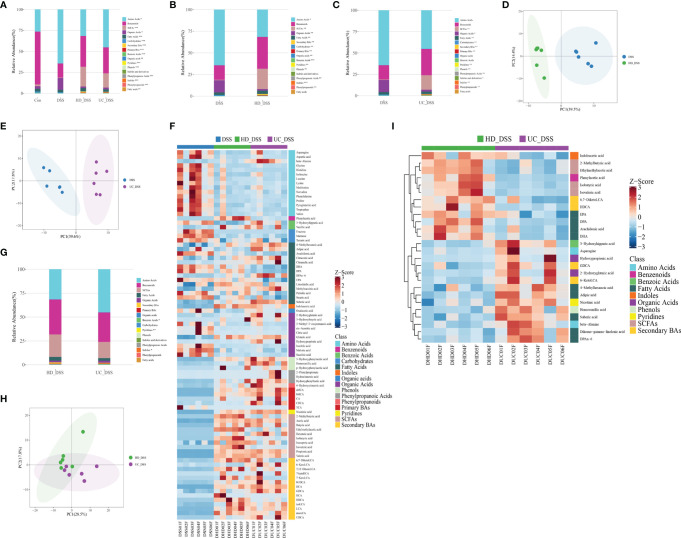
FMT significantly modulated the metabolism of DSS-induced colitis mice. **(A)** Overview of the composition of the metabolites in the four groups; **(B)** Overview of HD+DSS VS DSS metabolism; **(C)** Overview of UC+DSS VS DSS metabolism; **(D)** PCA of HD+DSS VS DSS; **(E)** PCA of UC+DSS VS DSS; **(F)** Biomarker heatmap of the DSS group, HD+DSS group and UC+DSS group (screening criteria are as follows: univariate statistics analysis P <0.05, |log2FC| >= 0 and multi-dimensional analysis VIP> 1); **(G)** Overview of metabolites in HD+DSS group and UC+DSS group; **(H)** PCA of HD+DSS VS UC+DSS; **(I)** Biomarker heatmap of HD+DSS group and UC+DSS group (screening criteria are as follows: single-dimensional analysis P <0.05, |log2FC| >= 0 and multi-dimensional analysis VIP> 1).

Given the prevalence of metabolic disorders in patients with IBD. We further studied the fecal metabolites of the four groups in order to elucidate the therapeutic mechanism of FMT, and identify the metabolic differences between FMT donors in the two groups with different effects. As shown in **Figures 7A–C**, intestinal bacteria metabolites SCFAs, SBAs, and indoles were significantly enriched in the two FMT groups, while amino acids, fructose, and other carbohydrates were significantly enriched in the control and DSS groups. Multi-dimensional statistics using principal component analysis (PCA), partial least-squares discrimination analysis (PLS-DA), orthogonal partial least-squares discrimination analysis (OPLS-DA) showed that the metabolic structure of HD+DSS group differed from that of the DSS group **(** **Figure 7D** and **Figure S1A** **)**, and that of the DSS group differed from that of the UC+DSS group **(** **Figure 7E** and **Supplementary Figure S1B** **)**. Based on the OPLS-DA model, volcano plot is helpful in selecting differential (statistically significantly changed) metabolites **(** **Supplementary Figure S1C**, DSS vs. HD_DSS; **Supplementary Figure S1D**, DSS vs. UC_DSS**).** In addition, differential metabolites between the DSS group and the HD+DSS group **(** **Supplementary Figure S1E** **)**, and between the DSS group and the UC+DSS group **(** **Supplementary Figure S1F** **)**, were screened using univariate statistical analysis and displayed using volcano plot. By obtaining union of the differential metabolites from univariate statistics and multi-dimensional statistics, potential biomarkers that may play critical roles in DSS-induced colitis were found in the three groups **(** **Figure 7F** **),** and anti-inflammatory SCFAs (e.g., butyric acid and acetic acid) and SBAs (e.g., LCA and DCA) were found to be abundant in both FMT groups.

It is necessary to analyze the metabolic differences between the HD+DSS and UC+DSS groups with different efficacies. The classification of metabolites is shown in **Figure 7G**; indoles were significantly enriched in the HD+DSS group, while benzoic acid and pyridine compounds were significantly enriched in the UC+DSS group. And although there were no statistically significant differences in the levels of SCFAs and SBAs between the two groups, the levels of SCFAs and SBAs in the HD+DSS group were higher. Multi-dimensional statistics using PCA, PLS-DA, and OPLS-DA showed that the metabolic structure of HD+DSS group differed from that of the UC+DSS group **(** **Figure 7H** and **Supplementary S1G** **)**, and volcano plot was helpful in identifying differential metabolites based on the OPLS-DA model **(** **Supplementary Figure S1H** **)**. The volcano plot of univariate statistics is shown in **Supplementary Figure S1I**. By obtaining intersection of the differential metabolites from univariate and multi-dimensional statistics, 26 potential biomarkers **(** **Supplementary Figure S1J** **)** that may play critical roles in DSS-induced colitis were identified and shown in a heatmap **(** **Figure 7I** **)**. Indoleacetic acid and SCFA metabolites (e.g., isovaleric acid, isobutyric acid, 2-Methylbutyric acid, ethylmethylacetic acid), SBAs metabolites HDCA and 6,7-DiketoLCA, Phenylacetic acid, and fatty acids metabolites (e.g., DHA, EPA, and DPA), were significantly enriched in the HD+DSS group, while amino acid metabolites asparagine and beta-alanine, pyridine metabolites nicotinic acid, SBAs metabolites 6,7-DiketoLCA and GDCA, fatty acid metabolite suberic acid, and pro-inflammatory n-6 PUFAs metabolite DPAn-6, were significantly enriched in the UC+DSS group. In summary, FMT significantly improved the metabolism in DSS-induced colitis, HD intervention was more effective than UC intervention, and the metabolisms of the two FMT groups were significantly different.

### Bacteria, Metabolites, Inflammatory Factors, and Intestinal Barrier Synergistically Regulate the Sensitivity to Mouse Colitis

**Figure 8:**
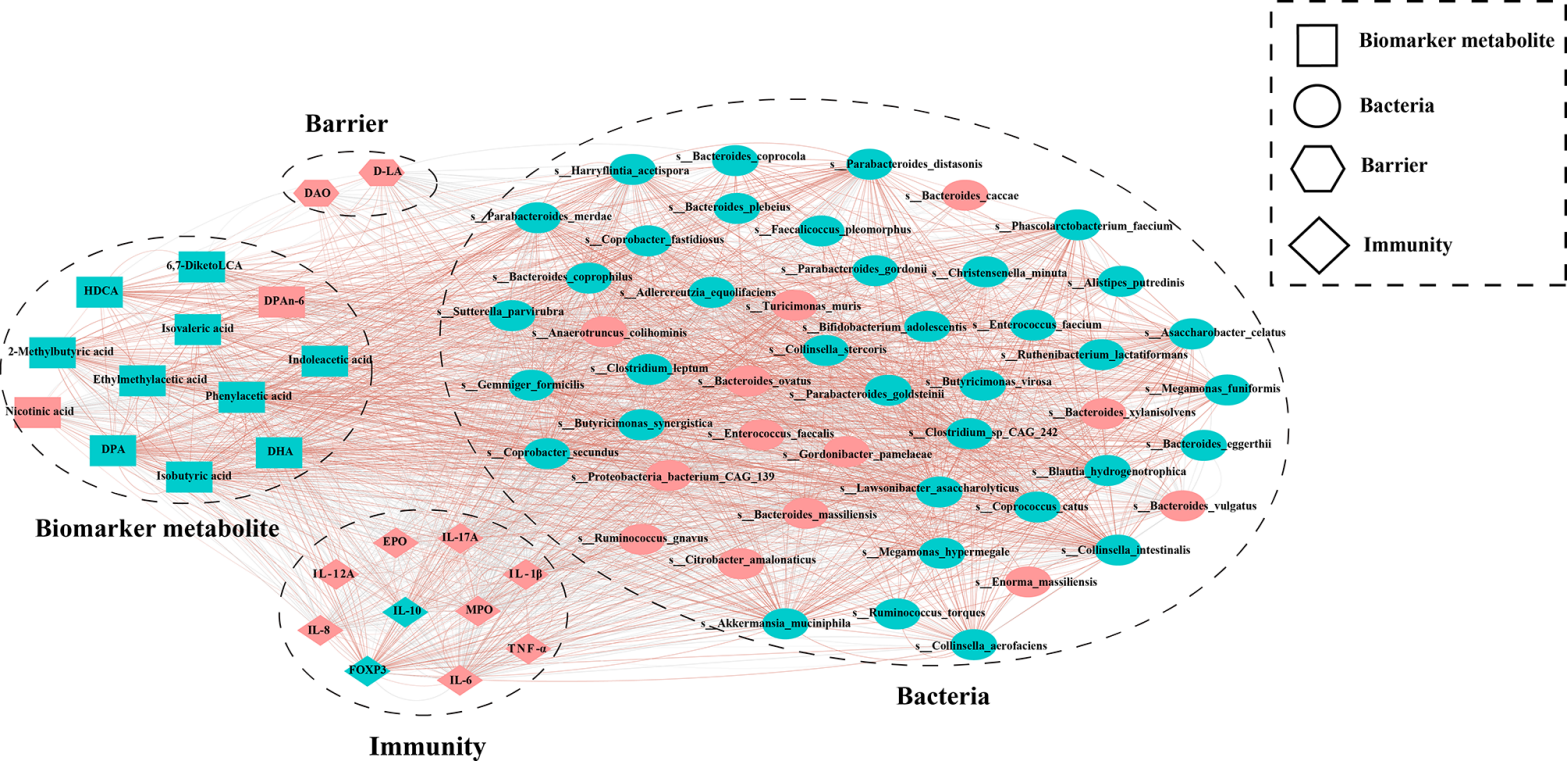
The co-occurrence analysis of differential bacteria, metabolites, intestinal barrier and immunity. The color of the nodes represents the enrichment group, Red represents enrichment in the UC_DSS group, Blue represents enrichment in the HD_DSS group. Edges between nodes indicate Spearman’s negative (light grey) or positive (light red) correlation; edge thickness indicates range of P value (P < 0.05).

To explore the potential correlation between different intestinal bacteria, metabolites, and inflammatory indicators, we conducted a co-occurrence analysis. Overall, co-occurrence analysis revealed a strong co-occurrence relationship between bacteria, metabolites, and inflammatory factors, and intestinal barrier **(** **Figure 8** **)**. In this co-expression network, bacteria, metabolites, inflammatory factors, and intestinal barrier, formed a total of 1197 robust correlations with themselves or with each other (Spearman correlation value > 0.5, p < 0.05). Among them, bacteria, metabolites, immunity, and intestinal barrier showed 517, 30, 29, and 1 correlations with themselves, respectively. In addition, there were 239, 30, and 269 correlations between bacteria and immunity, intestinal barrier, and metabolites, respectively. There were 5 and 72 correlations between immunity and intestinal barrier, and metabolites, respectively. And there were 5 correlations between metabolites and intestinal barrier. Therefore, these characteristic co-expression networks indicate that bacteria and metabolites are significantly related to inflammatory factors and the intestinal barrier, and the robust co-occurrence relationship regulates host sensitivity to colitis.

## Discussion

In this study, the effect of FMT intervention on IBD was explored using a DSS-induced GF mouse colitis model. The research data indicated that HD intervention significantly alleviated DSS-induced colitis, as evidenced by the prevention of body weight loss, decreased DAI and histology score, downregulated the levels of colitis markers MPO ( 30, 31 ) and EPO ( 32 ), and regulated cytokine homeostasis by providing more types of anti-inflammatory bacteria and metabolites. In addition, our data demonstrated that the UC intervention partially alleviated these symptoms, with less efficacy than HD intervention. Moreover, our data indicated a very broad and strong association between intestinal bacteria, metabolites, inflammatory factors, and the intestinal barrier. This co-occurrence regulated the susceptibility of GF mice to DSS.

The functional imbalance and uncontrolled expression of pro-inflammatory factors in patients with IBD promote disease progression ( 33 ). In this study, HD intervention significantly inhibited the expression of DSS-induced pro-inflammatory factors and had a better anti-inflammatory effect, while UC exerted anti-inflammatory effects by decreasing the levels of IL-6 and IL-12A. In addition, the levels of “protective cytokine” IL-10 increased significantly in the HD+DSS group, while only the serum IL-10 levels increased in the UC+DSS group. Foxp3 is involved in the development of Treg cells that secrete IL-10 to inhibit inflammation, and DSS-induced colitis results in a decrease in Foxp3^+^Treg cells ( 34 ). Compared to the DSS group, the two FMT intervention groups had significantly higher Foxp3 levels, and the HD+DSS group had higher Foxp3 levels than the UC+DSS group, which also explains why the HD intervention group had higher IL-10 levels. Uncontrolled expression of inflammatory factors can damage the intestinal barrier ( 35 ), and its dysfunction is linked to IBD ( 36 ). The expression of ZO-1 was reduced in colon biopsy samples from patients with IBD ( 37 ), and occludin expression was impaired by intestinal inflammation ( 38 ). In this study, compared to the DSS group, the two intervention groups showed significantly higher expression of ZO-1 in the colon, and the HD+DSS group had the highest ZO-1 levels. There was no difference in occludin levels between the UC+DSS and DSS groups, while the HD+DSS group had significantly higher levels of occludin in the mouse colon. Measurements of D-LA and DAO, which represent intestinal permeability ( 39, 40 ), suggested that the HD+DSS group had lower intestinal permeability. In summary, compared to UC intervention, HD intervention could better regulate the homeostasis of cytokines to improve the intestinal barrier in DSS-induced colitis mice.

The gut microbiota plays a key role in the initiation and progression of IBD ( 34 ). In this study, compared to the UC+DSS group the α-diversity of the microbial species composition was found to be higher in the HD+DSS group. IBD-related and pro-inflammatory microorganisms such as *Bacteroides vulgatus*, *Ruminococcus gnavus*, *Gordonibacter pamelaeae*, *Enterococcus faecalis*, *Bacteroides massiliensis*, and *Bacteroides xylanisolvens*, were enriched in the UC+DSS group. The relative abundances of *B. vulgatus* has been found to be directly correlated with elastase activity, which was significantly increased in patients with UC ( 41 ). In this study, MPO and pro-inflammatory factors were directly correlated with the relative abundance of *B. vulgatu*s. *R. gnavus*, *G. pamelaeae*, and *E. faecalis*. And these microorganisms were enriched in patients with IBD ( 13, 42–46 ). Similarly in this study, these microorganisms were significantly enriched in the UC+DSS group, and their relative abundances were significantly directly correlated with a variety of pro-inflammatory factors. The relative abundance of *B. massiliensis*, which has a pro-inflammatory effect ( 47 ) was significantly inversely correlated with IL-10 and Foxp3 levels, and significantly directly correlated with the levels of MPO, EPO, and a variety of pro-inflammatory factors such as IL-1β and TNF-α. It has been reported that colonization of GF mice with *B. xylanisolvens* could induce intestinal inflammation ( 48 ), and the relative abundance of *B. xylanisolvens* was directly correlated with the levels of MPO, EPO, and a variety of pro-inflammatory factors in this study. The phylum Proteobacteria is more abundant in patients with IBD ( 49 ). In this study, the abundance of *Proteobacteria bacterium CAG 139*, a taxonomic group of Proteobacteria, was directly correlated with the levels of MPO, EPO, DAO, D-LA, and a variety of pro-inflammatory factors, but significantly inversely correlated with IL-10 levels. In addition, some microorganisms that maintain intestinal health were enriched in the feces of the UC+DSS group, such as *Parasutterella excrementihominis*, *Blautia coccoides*, and SCFA-producing bacteria *Bacteroides stercoris* and *Blautia coccoides* ( 50, 51 ), which helped UC intervention reduce DSS-induced colitis sensitivity in mice. In addition, *Bacteroides intestinalis* and *Enorma massiliensis* were enriched in the UC+DSS group, and the mechanism of the enrichment needs to be explored.

*Akkermansia muciniphila* (AKK), *Bacteroides eggerthii, Bacteroides plebeius, Bifidobacterium adolescentis, Bacteroides ovatus*, and Parabacteroides goldsteinii, which have the potential to treat or alleviate IBD ( 34, 52–54 ), the SCFA-producing *bacterium Christensenella minuta* ( 55 ), and SBA-producing *bacterium Clostridium leptum* ( 56 ), were enriched in the HD+DSS group. It has been reported that probiotic AKK is potentially useful in the treatment of intestinal inflammatory diseases ( 52 ); in this study, a high relative abundance of AKK was significantly directly correlated with Foxp3, and inversely correlated with various pro-inflammatory factors. *B. adolescentis* improves DSS-induced colitis by stimulating protective Treg/Th2 responses ( 34 ). Moreover, *B. eggerthi, B. plebeius, B. ovatus*, and *P. merdae*, have been reported to be related to the remission of IBD symptoms ( 53, 54, 57 ). Consistently, these bacteria were significantly enriched in the HD+DSS group and negatively correlated with pro-inflammatory factors. The butyrate-producing bacterium C. minuta has strong immunoregulatory properties that can prevent intestinal damage in acute colitis ( 55 ). *P. goldsteinii* reduces intestinal inflammation by increasing the activity of mitochondria and ribosomes in colonic cells, and restoring amino acid metabolism ( 58 ). The SCFA-producing bacteria *Butyricimonas virosa*, Blautia hydrogenotrophica, and *Butyricimonas synergistica*, also showed a positive correlation with anti-inflammatory factors and a negative correlation with pro-inflammatory factors. *C. leptum* alleviates intestinal inflammation through a process that depends on the TGR5 bile acid receptor ( 56 ). In this study, the relative abundance of *C. minuta, P. goldsteinii*, and *C. leptum* were inversely correlated with a variety of pro-inflammatory factors and DAO and D-LA, which may contribute to the improved integrity of the intestinal barrier in the HD+DSS group. Parabacteroides distasonis is involved in the metabolism of bile acids, increases the level of host lithocholic acid (LCA) ( 59 ), and relieves the symptoms of IBD by regulating immune cells and reducing pro-inflammatory factors ( 16 ). *P. distasonis* was significantly enriched and negatively correlated with MPO, EPO, and a variety of pro-inflammatory factors in the HD+DSS group. We also found that *Alistipes finegoldii* and *Alistipes putredinis*, which represent the core characteristics of health status ( 60 ), were significantly enriched in the HD+DSS group, and negatively correlated with pro-inflammatory factors and intestinal injury indicators. *Coprobacter fastidiosus* and *Lawsonibacter asaccharolyticus* were positively correlated with IL-10, and their anti-inflammatory mechanism needs to be further explored.

In this study, the two FMT donors significantly altered the metabolism in colitis mice, and increased the levels of SCFAs, SBAs, and indole and its derivatives. SCFAs (mainly acetate, propionate, and butyrate) produced by intestinal bacteria, regulate protective immunity and reduce inflammation ( 61, 62 ). The SBAs enriched in the FMT group, such as LCA and UDCA, regulate immune cells and reduce the levels of pro-inflammatory factors, relieving IBD symptoms ( 56 ). Indoleacetic acid produced by tryptophan metabolism of intestinal bacteria can act as an agonist of AhR, which responds to numerous developmental and tissue-dependent effects on T cell immunity, and plays a protective and anti-inflammatory role in the intestinal tract through IL-22 ( 63 ). Card9^-/-^ mice, which are more susceptible to colitis, have a reduced ability to activate AhR, which is associated with decreased indole acetic acid levels ( 64 ). In this study, FMT significantly increased the indoleacetic acid levels in mouse feces, and to a greater extent in the HD+DSS group than in the UC+DSS group, which helped the HD+DSS group exert a better effect. In addition, the unsaturated fatty acids DPA, EPA, and DHA, were significantly enriched in the HD+DSS group. It has been noted that EPA and DHA have anti-inflammatory effects in inflammation models ( 65 ). However, DPAn-6, with a significant pro-inflammatory effect, was enriched in the UC+DSS group ( 66 ). Nicotinuric acid was significantly enriched in the feces of mice in the UC+DSS group, and while it was found to be almost exclusively present in the feces of patients with IBD ( 44 ), the cause of nicotinic acid enrichment needs to be investigated. In summary, FMT modulated metabolism in DSS-induced colitis mice, and HD intervention was more effective than UC intervention.

The results of this study indicated that UC intervention could slightly alleviate the symptoms of colitis, as evidenced by the presence of anti-inflammatory microorganisms (*P. excrementihominis*, *B. coccoides*, *B. stercoris*, etc.) and metabolites (SCFAs, SBAs, indole, and its derivatives) in the feces of UC+DSS group mice. This explains why the UC bacteria have anti-inflammatory effects. Moreover, our data indicated that there were more kinds of beneficial microorganisms and metabolites in the HD+DSS group than in the UC+DSS group, which clearly explains the better efficacy of HD intervention in alleviating DSS-induced colitis from a microbiological perspective.

In conclusion, these results indicate that HD intervention can regulate cytokine homeostasis by providing more types of anti-inflammatory bacteria and metabolites, protecting the intestinal mucosal barrier, and significantly reducing the sensitivity to DSS-induced colitis, compared to the UC+DSS group. It is noteworthy that the UC bacteria considered to be “deteriorated” also reduced the sensitivity of GF mice to DSS to a certain extent. This suggests that the bacteria, metabolites, inflammatory factors, and intestinal barrier, can regulate the sensitivity of mice to DSS-induced colitis through a strong and extensive synergistic relationship. This study provides new insights into the microecological interventions for IBD.

## Data Availability Statement

The datasets presented in this study can be found in online repositories. The names of the repository/repositories and accession number(s) can be found below: NMDC (https://nmdc.cn/). The accession number NMDC40014687.

## Ethics Statement

The animal study was reviewed and approved by The Tab of Animal Experimental Ethical Inspection of Laboratory Animal Centre, Huazhong Agriculture University.

## Author Contributions

YY, ST and HW designed the experiment. YY, XJZ, YW, XT, HCZ, SF, HZ, ZZ, JH, BC, XYZ, ZW, MD, WC performed the animal trials, and sample and data analysis. YY drafted the manuscript. ST and HW revised the manuscript. All authors contributed to the article and approved the submitted version.

## Funding

This work was supported by the National Key Research and Development Program of China (2018YFC2000504), the National Nature Science Foundation of China (31902189), the Natural Science Foundation of Hubei Province (2021CFB436; 2021CFA018), and the Fundamental Research Funds for the Central Universities (2662020DKQD004).

## Conflict of Interest

The authors declare that the research was conducted in the absence of any commercial or financial relationships that could be construed as a potential conflict of interest.

## Publisher’s Note

All claims expressed in this article are solely those of the authors and do not necessarily represent those of their affiliated organizations, or those of the publisher, the editors and the reviewers. Any product that may be evaluated in this article, or claim that may be made by its manufacturer, is not guaranteed or endorsed by the publisher.

## Supplementary Material

Supplementary Figure 1 HD intervention significantly modulated the metabolism of DSS-induced colitis mice. **(A)** OPLS-DA and PLS-DA of HD+DSS VS DSS; **(B)** OPLS-DA and PLS-DA of UC+DSS VS DSS; **(C)** Differential metabolites between HD+DSS group and DSS group were obtained using Multi-Dimensional Statistics; **(D)** Differential metabolites between UC+DSS group and DSS group were obtained using Multi-Dimensional Statistics; **(E)** Differential metabolites between HD+DSS group and DSS group were obtained using univariate statistical analysis (student T-test or Mann-Whitney U test, depending on the normality of data and homogeneity of variance) (screening criteria are as follows: univariate statistics analysis P < 0.05, |log2FC| >= 0); **(F)** Differential metabolites between UC+DSS group and DSS group were obtained using univariate statistical analysis (student T-test or Mann-Whitney U test, depending on the normality of data and homogeneity of variance) (screening criteria are as follows: univariate statistics analysis P < 0.05, |log2FC| >= 0); **(G)** OPLS-DA and PLS-DA of HD+DSS VS UC+DSS; **(H)** Differential metabolites between HD+DSS group and UC+DSS group were obtained using Multi-Dimensional Statistics; **(I)** Differential metabolites between HD+DSS group and UC+DSS group were obtained using univariate statistical analysis (student T-test or Mann-Whitney U test, depending on the normality of data and homogeneity of variance) (screening criteria are as follows: univariate statistics analysis P < 0.05, |log2FC| >= 0); **(J)** Venn Plot of differential metabolites.
