# Supplementary figures and images for: Human Fecal Microbiota Transplantation Reduces the Susceptibility to Dextran Sulfate Sodium-Induced Germ-Free Mouse Colitis
Source: Front Immunol. 2022 Feb 14;13:836542. doi: 10.3389/fimmu.2022.836542 (PMC8882623; doi:10.3389/fimmu.2022.836542)

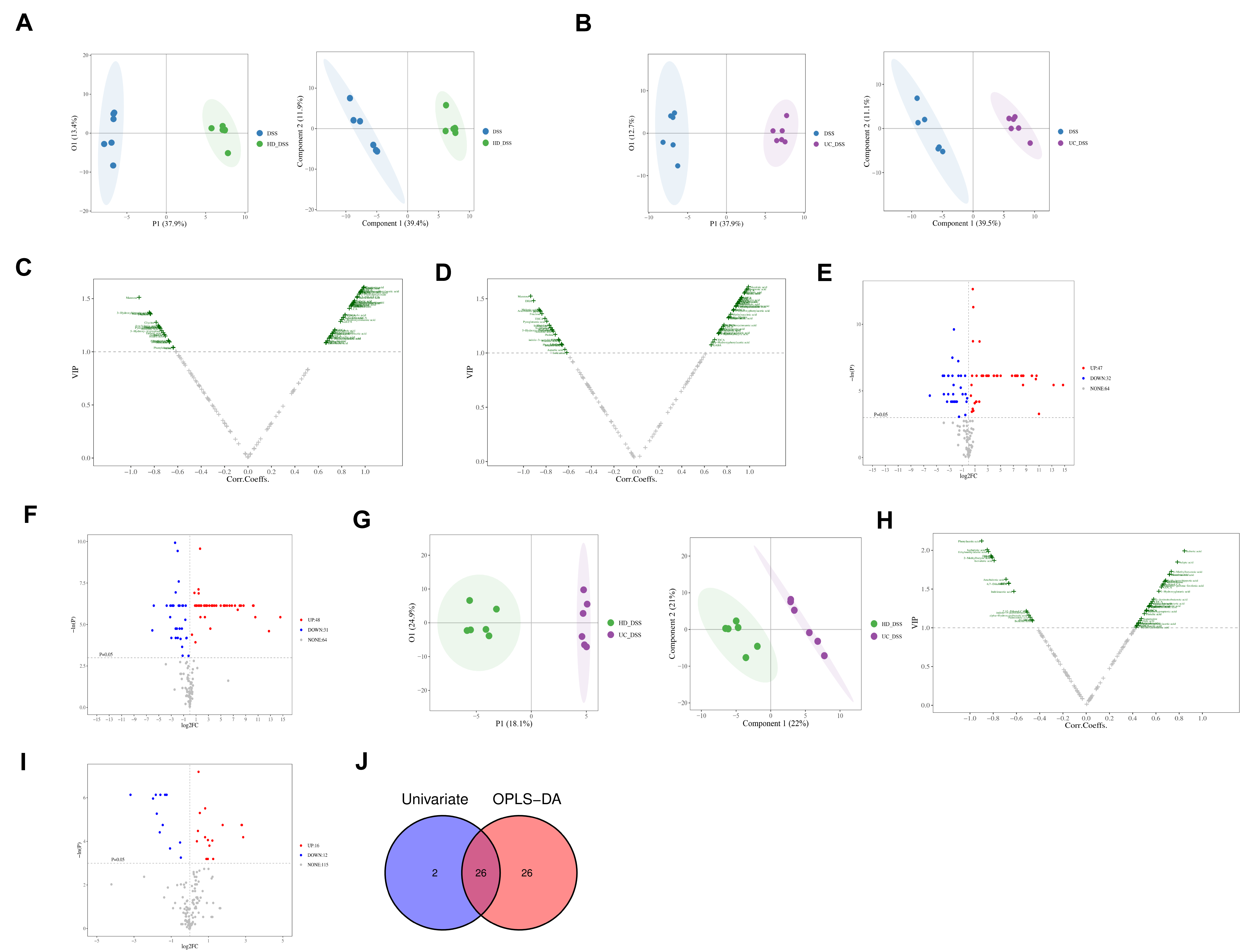

Supplement: Supplementary Figure 1 — HD intervention significantly modulated the metabolism of DSS-induced colitis mice. (A) OPLS-DA and PLS-DA of HD+DSS VS DSS; (B) OPLS-DA and PLS-DA of UC+DSS VS DSS; (C) Differential metabolites between HD+DSS group and DSS group were obtained using Multi-Dimensional Statistics; (D) Differential metabolites between UC+DSS group and DSS group were obtained using Multi-Dimensional Statistics; (E) Differential metabolites between HD+DSS group and DSS group were obtained using univariate statistical analysis (student T-test or Mann-Whitney U test, depending on the normality of data and homogeneity of variance) (screening criteria are as follows: univariate statistics analysis P < 0.05, |log2FC| >= 0); (F) Differential metabolites between UC+DSS group and DSS group were obtained using univariate statistical analysis (student T-test or Mann-Whitney U test, depending on the normality of data and homogeneity of variance) (screening criteria are as follows: univariate statistics analysis P < 0.05, |log2FC| >= 0); (G) OPLS-DA and PLS-DA of HD+DSS VS UC+DSS; (H) Differential metabolites between HD+DSS group and UC+DSS group were obtained using Multi-Dimensional Statistics; (I) Differential metabolites between HD+DSS group and UC+DSS group were obtained using univariate statistical analysis (student T-test or Mann-Whitney U test, depending on the normality of data and homogeneity of variance) (screening criteria are as follows: univariate statistics analysis P < 0.05, |log2FC| >= 0); (J) Venn Plot of differential metabolites. [file Image_1.tif]
